# The Enigmatic Presentation of Pneumorrhachis in Necrotizing Soft Tissue Infection: A Case Report

**DOI:** 10.7759/cureus.96032

**Published:** 2025-11-03

**Authors:** Haitham Siag, Zana J Hanini, Vance Smith

**Affiliations:** 1 Department of Surgery, Montefiore New Rochelle Hospital, New Rochelle, USA; 2 Department of Surgery, Montefiore Einstein Medical Center, Bronx, USA

**Keywords:** epidural abscess, general neurosurgery, general surgery, interventional radiology, necrotizing soft tissue infection, nsti, pan myelomeningitis, pneumorrhachis, radiology, spinal neurosurgery

## Abstract

Pneumorrhachis, the presence of air within the spinal canal, is an uncommon radiologic finding, most often resulting from trauma or iatrogenic procedures, or exceedingly rarely, infectious causes. We present a case of necrotizing soft tissue infection overlying the sacrum, not associated with a preexisting pressure ulcer/wound, leading to pneumorrhachis, pan myelomeningitis, and epidural abscess formation. Initial diagnosis was assumed to be a buttock abscess, but additional symptoms of severe weakness and lethargy elicited further studies. Prompt diagnosis was achieved with computed tomography, magnetic resonance imaging, and operative intervention, leading to a more favorable outcome. This case exemplifies the need to maintain high awareness for seemingly mundane symptoms, as they may mask more serious pathologies like pneumorrhachis or epidural abscess.

## Introduction

Pneumorrhachis is a rare condition characterized by gas within the spinal canal, also known as epidural emphysema, aerorachia, and intraspinal air [1]. The most common causes of pneumorrhachis are traumatic or iatrogenic, and rarely infectious origins [1,2]. In the appropriate clinical setting, vigilance must be exercised to diagnose and address the condition promptly and effectively, thereby limiting its progression and the resulting morbidity [1,3].

In this case, we describe the swift diagnosis and management sequence of what was initially thought to be cellulitis with an underlying abscess but was found to be necrotizing soft tissue infection (NSTI) invading the spinal column, causing pneumorrhachis, myelomeningitis, and an epidural abscess not associated with a chronic wound, i.e., a pressure ulcer [4-6]. Due to the condition's rarity, it is challenging to delineate a particular incidence, which makes for an exciting case discussion regarding when to escalate investigations versus stay the course and treat empirically [1,7,8].

## Case presentation

The patient was a 63-year-old male with a past medical history of hypertension and diabetes mellitus type 2, who was brought in due to a decrease in mental state and recurrent uncontrolled fevers for two days. Upon further history, it was noted that the patient was complaining of buttock pain three days before presentation, which progressed into swelling and pain in the area. The family further noticed that the patient had an abnormal gait, difficulty ambulating, generalized weakness, and fatigue.

The patient initially presented with a temperature of 105° Fahrenheit, a heart rate of 132 beats per minute, and was normotensive. On exam, he had a notable induration and firm swelling of the right medial buttock. Neurological exam revealed that he was responsive to vigorous stimuli only; otherwise was unremarkable. Due to the patient’s presentation, the emergency department initiated a sepsis protocol, with appropriate antibiotic coverage, including vancomycin and piperacillin-tazobactam. At that time, the differential diagnoses were COVID, pneumonia, or a large buttock abscess with severe cellulitis. The decision was made to obtain a Computed Tomography (CT) scan to ascertain the extent of the possible abscess.

Upon surgical evaluation within two hours, examination was notable for positive Brudzinski’s sign, with intact skin. The surgical team recommended getting a C-reactive protein level, which resulted in 247.4 mg/dL, prompting broadening of the antibiotic coverage with clindamycin.

**Figure 1:**
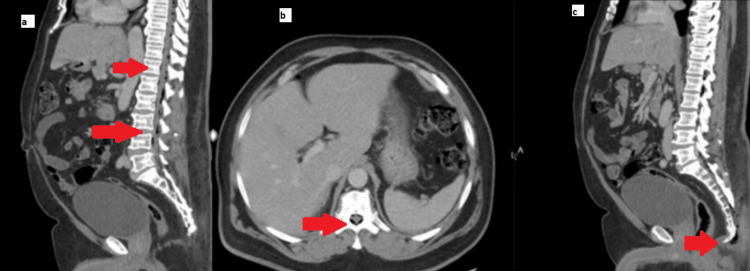
Computed tomography scan showing extensive soft tissue air extending into the spinal canal with multiple air foci in the epidural space of the thoracolumbar spine, concerning for sacrococcygeal infection/inflammation extending through the spinal canal, recommend Magnetic Resonance Imaging to exclude epidural intraspinal abscess. (A) Sagittal image, extensive soft tissue air extending into the spinal canal with multiple air foci in the epidural space of the thoracolumbar spine (B) Axial image, showing air within the spinal column (C) Sagittal image, subcutaneous tissue stranding with multiple air foci at the sacrococcygeal region

Due to the severe and life-threatening condition of the patient, transfer to a higher-level facility was recommended, as the availability of neurosurgery became paramount. Pertinent labs are shown in Table 1, and the initial CT is shown in Figure 1.

**Table 1:** Labaratory values for the patient upon initial presentation

Name	Value	Reference Range
White Blood cells	23.3 x 10*3/ul	4.8 - 10.8 10*3/ul
Hemoglobin	13.4 g/dl	14.0 - 17.4 g/dL
Serum Lactate	2.4 mmol/l	0.5 - 2.0 mmol/l
Serum Blood Glucose	326 mg/dl	70 - 140 mg/dl
Serum Creatinine	1.54 mg/dl	0.50 - 1.20 mg/dl
Serum Sodium	131 mmol/L	135 - 145 mmol/L
C-Reactive Protein	247.4 mg/dL	<0.8 mg/dL

**Figure 2:**
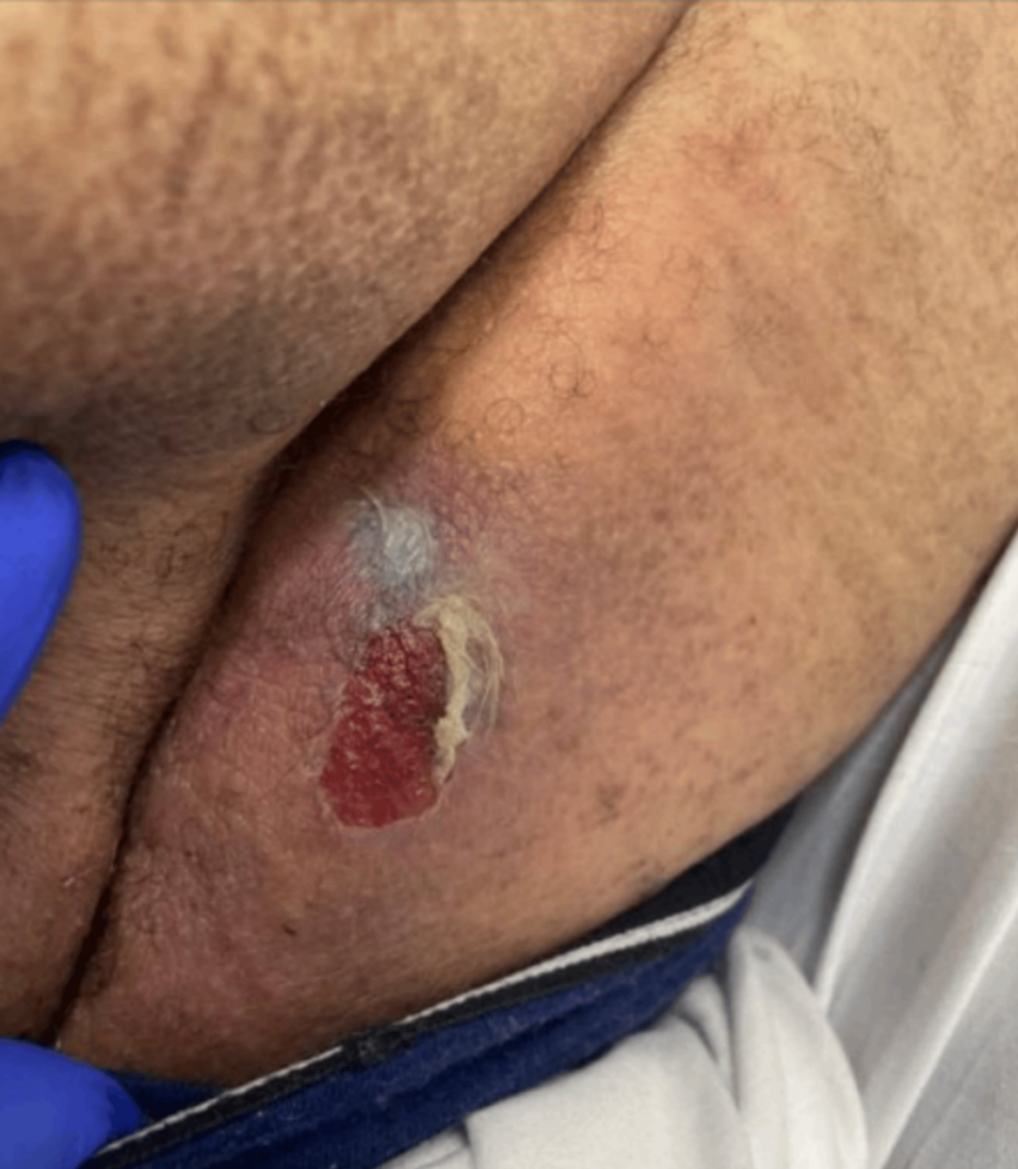
A small area of skin sloughing with exposed dermis and associated induration but no crepitus

**Figure 3:**
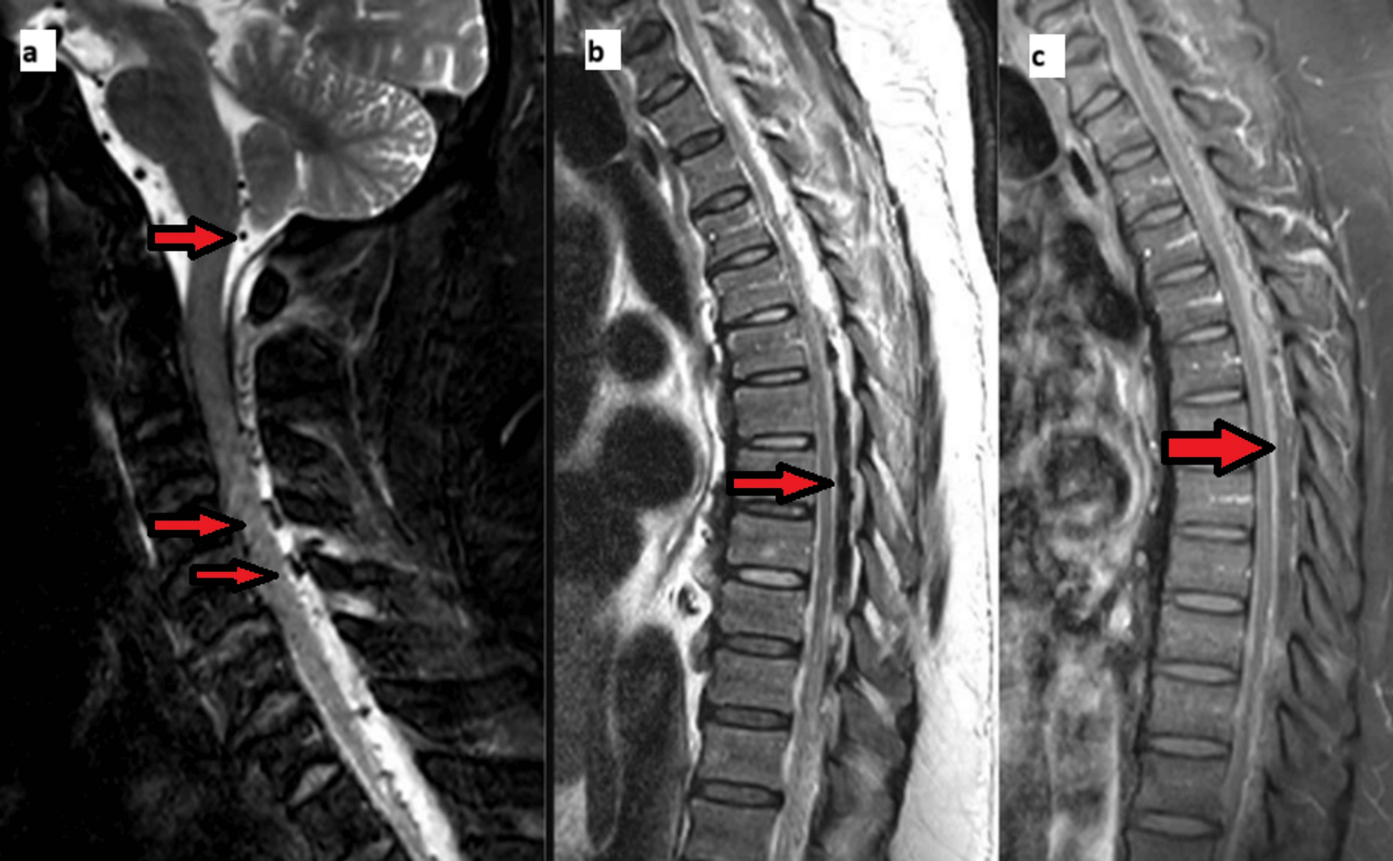
Magnetic resonance sagittal images showing long segment epidural fluid collection extending from the level of the foramen magnum to the visualized upper cervical spine, edema within the posterior neck soft tissues, left greater than right, long segment epidural fluid collection which extends the entire level of the spinal cord from the level of foramen magnum through the sacral canal. Heterogeneous epidural soft tissue signal intensity and enhancement is seen, with more localized air collection in the mid thoracic spine, diffuse enhancement of the epidural space and spinal dura, compatible with inflammatory change. (A) Dark signal on T1-weighted magnetic resonance imaging denotes foci of air (B) Long segment epidural fluid collection which extends the entire level of the spinal cord from the level of foramen magnum through the sacral canal. Heterogeneous epidural soft tissue signal intensity and enhancement is seen, with more localized air collection in the mid thoracic spine * (C) Long segment mass effect and compression of the CSF spaces within the thecal sac with anterior displacement of the spinal cord. Is greatest in the mid thoracic spine, with suspected focal signal changes within the cord at the level of T6.

Upon presentation to the tertiary-level care center, the skin exam had changed, as shown in Figure 2. The infectious disease team was consulted, and meropenem and linezolid were recommended for coverage of possible polymicrobial origin, namely Streptococcus anginosus or other group A Streptococcus species as the patient was not in septic shock. Cultures later revealed Actinomyces radingae, Actinotignum shaali, and Finegoldia, which are part of normal skin flora. The neurosurgery team was consulted, and a Magnetic Resonance Imaging was obtained, as shown in Figure 3.

Due to a lack of significant spinal cord compression (despite material within the canal), the neurosurgery team decided to defer a spinal decompression procedure as there was extensive spread of the infection from the occiput to the lower sacrum; they believed that source control or evacuation would not be possible, and likely too morbid for the patient.

**Figure 4:**
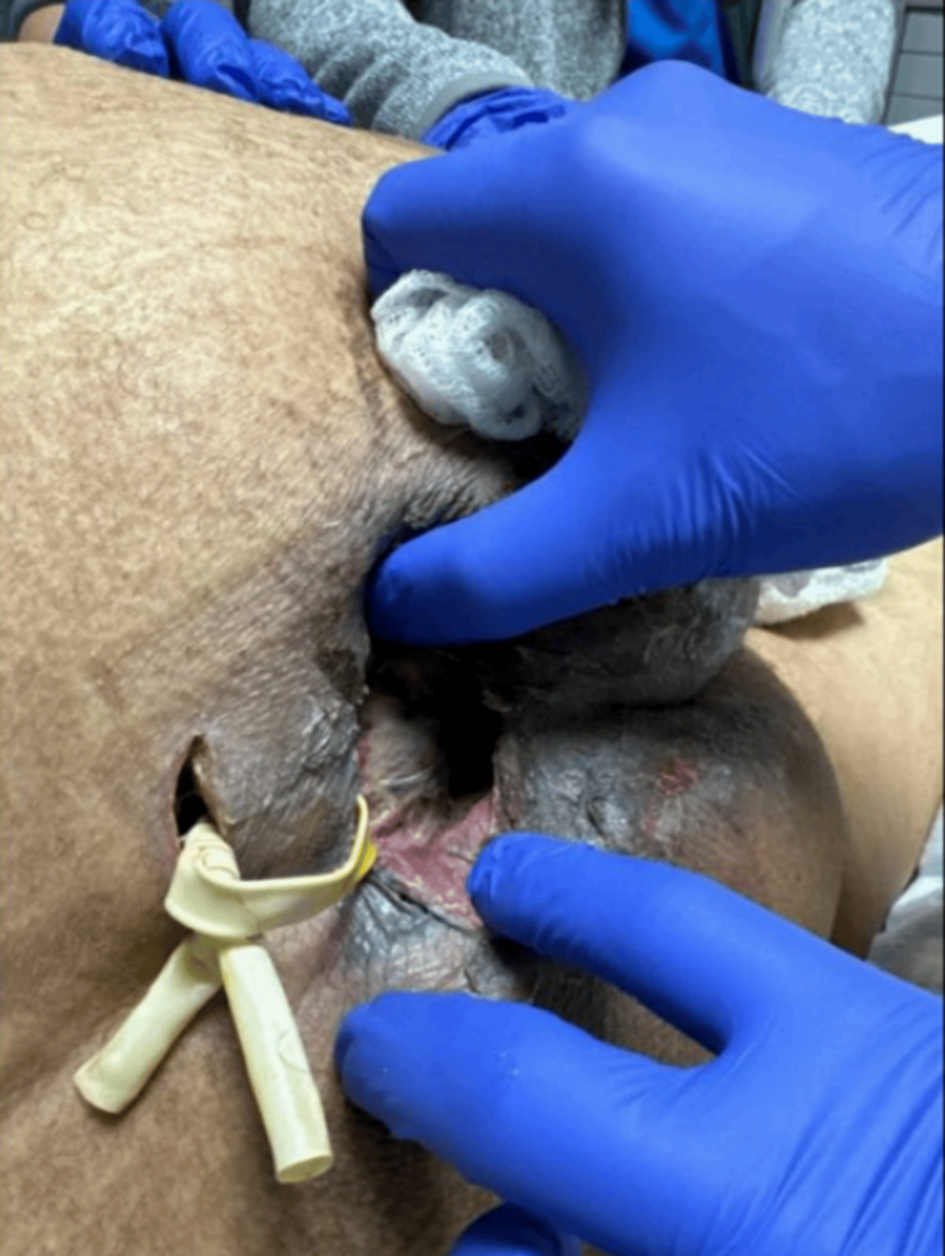
Surgical wound, showing the Penrose drain.

**Figure 5:**
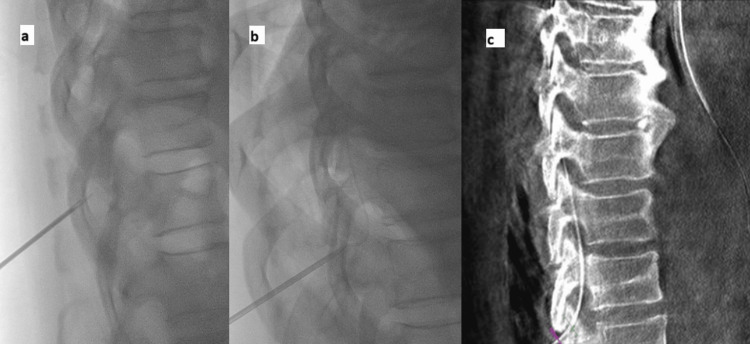
Interventional Radiology drain placement under fluoroscopy (A) showing the needle placement (B) showing needle with insertion of guidewire (C) final drain placement

The patient was subsequently taken to the operating room for incision and drainage with local debridement of the sacral region. Findings during the procedure were necrotic tissue with purulent expression, debrided down to the periosteum of the sacrum, and noted one tracking sinus deep to the spine. A counter incision was made, and a Penrose drain was placed, as seen in Figure 4. Neurological Interventional Radiology was consulted on 1/8/2024, and radiologic-directed drainage of the epidural abscess was placed via fluoroscopy as seen in Figure 5. This drain was then removed two days later.

The subsequent hospital course was complicated by multiple cardiac arrests and bradycardic episodes, resulting in a semi-permanent pacemaker, a seizure episode, and ultimately resulting in a tracheostomy and percutaneous endoscopic gastrostomy tube. The patient was subsequently discharged several months after initial presentation.

## Discussion

Pneumorrhachis on imaging should always alert us to a more serious diagnosis, as pneumorrhachis is the presence of intraspinal air [1]. This is more likely to be traumatic or iatrogenic in most cases; however, the rare instances of infection, as in our case, require a more diligent workup [2].

Determining the primary cause of pneumorrhachis should begin with a CT scan, and, depending on the associated signs and symptoms, may be followed by a Magnetic Resonance Imaging to further delineate the pathology [1]. This sequential imaging approach ensures accurate identification of the underlying condition, such as infection or trauma, guiding appropriate treatment. Its presence could be an asymptomatic epiphenomenon, causing pressure symptoms (pain, discomfort, muscle weakness, headache, neurological deficits, i.e., cauda equina or acute nerve root compression), or having symptoms related to the underlying condition [3]. Treatment options for uncomplicated pneumorrhachis include oxygen therapy with inpatient observation in asymptomatic or mild cases. Rare cases require decompressive surgery or other drainage procedures, such as in our case [1].

This case illustrates the importance of swift diagnosis and intervention to manage necrotizing soft tissue infection, particularly when it invades the spinal column, resulting in severe complications such as the radiologic finding of pneumorrhachis, leading to the diagnosis of myelomeningitis and epidural abscess [2]. The sequence of events described required speedy diagnosis, transfer to a tertiary-level care center with neurosurgical and neuro-interventional capabilities, and the availability of Magnetic Resonance Imaging to appropriately analyze the presence and treatment of the epidural abscess [4].

Notably, the rarity includes the presence of three highly uncommon microbial pathogens (skin flora) as the cause of the necrotizing soft tissue infection: Actinomyces radingae, Actinotignum schaalii [5], and Finegoldia [6].

Due to the patient experiencing numerous complications, the unusual presentation of pneumorrhachis, myelomeningitis, and epidural abscess due to necrotizing soft tissue infection carried a compounded high level of mortality. Literature offers mortality rates for necrotizing soft tissue infection ranging from 15% to 30%, up to 100% in cases of existing comorbidities [7]. Further complications like epidural abscesses contribute to the risk of necrotizing soft tissue infection, with mortality rates at 14% [4]. For necrotizing soft tissue infection, although the recommended imaging modality is CT, due to its sensitivity and specificity exceeding 90%, Magnetic Resonance Imaging is the preferred modality for evaluating necrotizing soft tissue infection and spinal involvement, with early debridement and drainage to improve the mortality rate [7]. Early surgical intervention is paramount, as evidenced by mortality rates of 19% in patients undergoing debridement within six hours compared to 32% for delayed debridement [8]. Furthermore, in cases of epidural abscess, early surgical management is associated with a lower all-cause mortality rate (13.07%) compared to medical management alone (16.22%) [4].

## Conclusions

Due to its rarity, we recommend a multidisciplinary approach in managing such cases, especially in the setting of infection, to streamline the diagnosis and management of pneumorrhachis. An image finding could denote a much more severe diagnosis at hand. The findings in this case underscore the benefits of aggressive and timely intervention in improving outcomes for such high-risk patients. No other cases of reported pneumorrhachis in the context of necrotizing soft tissue infection were found in a comprehensive literature review.

Further research is warranted to better understand the pathophysiological mechanism of pneumorrhachis in various settings, including necrotizing soft tissue infection.
